# Proper Food Keeps off Disease

**Published:** 1919-03

**Authors:** 


### Proper Food Keeps off Disease.

All doctors know that a large portion of the sickness of the people is due directly to improper eating. Some people eat too much, some do not eat enough, some eat foods that are poorly adapted to their conditions, some eat things that are positively harmful. More and more, careful physicians are ascertaining what their patients eat and are prescribing their food just as they do their medicines. They know that proper food wards off disease, while medicine is only a curative agency.

Hereafter the family doctor is going to be a counselor in all things pertaining to the health of the family, and he will be consulted more frequently as to the effects of the foods the people eat. The new era in medical practice is tending toward prevention rather than toward the cure of ills, and those physicians who study food values and have the courage to say what people shall or shall not eat will be the popular family medical advisers.

